# Chromogenic Multiplex Immunohistochemistry Reveals Modulation of the Immune Microenvironment Associated with Survival in Elderly Patients with Lung Adenocarcinoma

**DOI:** 10.3390/cancers10090326

**Published:** 2018-09-13

**Authors:** Marius Ilié, Mélanie Beaulande, Saima Ben Hadj, Emmanuel Chamorey, Renaud Schiappa, Elodie Long-Mira, Sandra Lassalle, Catherine Butori, Charlotte Cohen, Sylvie Leroy, Olivier Guérin, Jérôme Mouroux, Charles-Hugo Marquette, Jean-François Pomerol, Gilles Erb, Véronique Hofman, Paul Hofman

**Affiliations:** 1 Laboratory of Clinical and Experimental Pathology, Université Côte d’Azur, CHU Nice, FHU OncoAge, Pasteur Hospital, 06000 Nice, France; ilie.m@chu-nice.fr (M.I.); long-mira.e@chu-nice.fr (E.L.-M.); lassalle.s@chu-nice.fr (S.L.); butori.c@chu-nice.fr (C.B.); hofman.v@chu-nice.fr (V.H.); 2 CNRS, INSERM, IRCAN, FHU OncoAge, Université Côte d’Azur, Team 4, 06000 Nice, France; 3 Hospital-Integrated Biobank (BB-0033-00025), CHU Nice, FHU OncoAge, Université Côte d’Azur, 06000 Nice, France; 4 EMEA-LATAM Division, Roche Diagnostics France, 38240 Meylan, France; melanie.beaulande@roche.com (M.B.); gilles.erb@roche.com (G.E.); 5 Imaging Analysis, Tribvn Healthcare, 92320 Châtillon, France; sbenhadj@tribvn-hc.com (S.B.H.); jfpomerol@tribvn-hc.com (J.-F.P.); 6 Biostatistics Unit, FHU OncoAge, Antoine Lacassagne Comprehensive Cancer Center, 06189 Nice, France; emmanuel.chamorey@nice.unicancer.fr (E.C.); Renaud.SCHIAPPA@nice.unicancer.fr (R.S.); 7 Department of Thoracic Surgery, FHU OncoAge, CHU Nice, Université Côte d’Azur, 06000 Nice, France; cohen.c@chu-nice.fr (C.C.); mouroux.j@chu-nice.fr (J.M.); 8 Department of Pulmonary Medicine and Thoracic Oncology, Pasteur Hospital, Université Côte d’Azur, CHU Nice, FHU OncoAge, 06000 Nice, France; leroy.s2@chu-nice.fr (S.L.); marquette.c@chu-nice.fr (C.-H.M.); 9 Department of Geriatric Medicine, Cimiez Hospital, Université Côte d’Azur, CHU Nice, FHU OncoAge, 06000 Nice, France; guerin.o@chu-nice.fr

**Keywords:** elderly, lung adenocarcinoma, multiplex immunohistochemistry, brightfield, immunosenescence

## Abstract

With underrepresentation of elderly patients with lung adenocarcinoma (LADC) in anti-PD-1/PD-L1 clinical trials, better understanding of the interplay of PD-L1 and tumor-associated immune cells (TAICs) could assist clinicians in stratifying these patients for immunotherapy. One hundred and one patients with LADCs, stratified by age, were included for analysis of PD-L1 expression and density of TAICs expressing CD4, CD8, and CD33, by using multiplex chromogenic immunohistochemistry (IHC) assays and automated digital quantification. The CD4^+^/CD8^+^ ratio was significantly higher in elderly patients. In patients <75 years, the density of CD4^+^, CD8^+^, and PD-L1 in TAICs showed a positive significant correlation with PD-L1 expression in tumor cells (TCs), while a lower correlation was observed in the elderly population. In the latter, a high CD4^+^/CD8^+^ ratio, and combined PD-L1 expression ≥1% TCs with a low CD8^+^ density, low CD33^+^ density, and a high CD4^+^ density correlated to worse overall survival. We identified differences according to age in the CD4^+^/CD8^+^ ratio and in correlation between PD-L1 expression and the density of TAICs in LADC patients. Distinct groups of tumor microenvironments had an impact on the OS of elderly patients with LADC.

## 1. Introduction

Therapies targeting programmed death 1 (PD-1) and programmed death ligand 1 (PD-L1) have demonstrated clinical improvement for some patients with immunogenic cancer types, including advanced lung adenocarcinoma (LADC) [1]. PD-L1 expression in tumors or tumor-associated immune cells (TAICs) assessed by immunohistochemistry (IHC) is the predictive biomarker evaluated in clinical trials, and is currently used in the clinical setting to select patients who may benefit from these therapies [2].

Despite the impressive clinical activity of PD-1/PD-L1 inhibitors in patients with advanced LADC, some patients do not benefit from this therapy, and the majority of those who respond develop resistance [3]. In this context, intense research is focusing on improving patient stratification for PD-1/PD-L1 immune-based therapy by optimizing the assessment of PD-L1 expression, and by identifying multiparametric, integrated immune-based biomarkers on formalin-fixed paraffin-embedded (FFPE) tissue sections [4,5,6].

Moreover, in a disease where approximately two-thirds of cases are diagnosed in patients aged over 65, and one-third of patients aged over 75, it is unclear if age affects patient responsiveness to immunotherapy [7,8]. Currently, no randomized phase III trials on the efficacy of PD-1/PD-L1 inhibitors in elderly patients with advanced non-small cell lung carcinoma (NSCLC) are available. Recent meta-analyses explored the differential efficacy of PD-1/PD-L1 inhibitors in old patients compared to young adults, raising the question of whether these treatments are effective in older patients [9,10]. There is only modest representation of older patients in pivotal clinical trials, and further assessment of the efficacy of immunotherapy in the elderly, specifically those representative of the average clinical trial–ineligible patient, is urgently needed [8,10].

Notably, aging is associated with remodeling of immune functions called “immunosenescence”, involving both innate and adaptive immunity [11]. Experimental animal and human studies have revealed a variety of age-associated changes, such as an extensive decrease in the number of naïve CD8^+^ T cells, which are associated with an increase in aged memory nonfunctional CD4^+^ T cells, upregulation of surface-expressed inhibitory receptors such as PD-1 with a decrease of the costimulatory molecules, higher concentration of inflammatory cytokines, a decreased number or impaired function of antigen-presenting cells such as dendritic cells and macrophages, qualitative alteration of natural killer and natural killer T cells, and the accumulation of regulatory cells such as regulatory T cells and immunosuppressive myeloid-derived suppressor cells (MDSCs) [12]. This phenomenon of immunosenescence could affect the efficacy and/or the toxicity of immune checkpoint blockade. Hyperprogressive behavior in patients treated with PD-1/PD-L1 inhibitors was associated in one study with an older age and with worse overall survival (OS) [13]. Therefore, factors other than PD-L1 expression, including tumor-infiltrating lymphocytes and tumor-associated MDSCs, may be influenced by aging, and thus, may drive variable responses to PD-1/PD-L1 inhibitors.

This study aimed to determine whether age influences the immunologically-defined LADC tissue microenvironment using quantitative chromogenic multiplex IHC, and to what extent these changes might exert a prognostic effect on LADC patients. We characterized the expression of PD-L1 in tumor and TAICs, and evaluated the density of TAICs expressing CD4, CD8, and CD33 on sequential whole tumor sections from resected stage II-IV LADCs. We performed quantitative digital image analysis, and correlated the findings with the patients’ clinical and pathological features in an age-stratified cohort of 101 patients with stage II-IV LADCs.

## 2. Results

### 2.1. Multiplex Immunohistochemical Assessment

**Figure 1:**
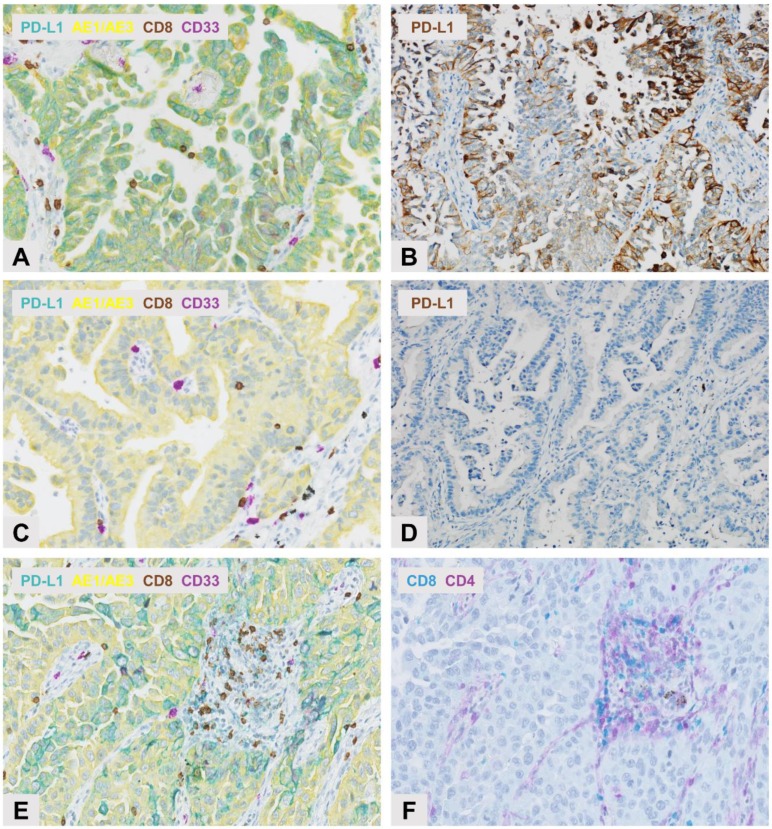
Multiplex immunohistochemistry (IHC) detection of programmed death ligand 1 PD-L1 expression and selected immune cell subpopulations in sequential whole tissue sections of lung adenocarcinoma (LADC). Upper panel: strong PD-L1 expression observed in the same LADC case following (**A**) PD-L1 (Teal) and AE1/AE3 (Yellow) chromogenic labeling showing the co-expression of PD-L1 and pan-Keratin AE1/AE3 in neoplastic cells, and (**B**) by immunoperoxidase (brown) with no clear distinction between PD-L1 labeling in tumor cells (TCs) or stromal cells. Original magnification, ×200. Middle panel: absence of PD-L1 expression observed in the same LADC case following (**C**) PD-L1 (Teal) and AE1/AE3 (Yellow) chromogenic labeling, and (**D**) with immunoperoxidase (brown). Original magnification, ×200. Lower panel: IHC detection of immune cell subpopulations in sequential sections of LADC following (**E**) Multiplex chromogenic detection by DAB staining (brown) of CD8^+^ lymphocytes and purple staining of CD33^+^ MDSCs, and (**F**) Teal immunolabeling of CD8^+^ lymphocytes and purple staining of CD4^+^ lymphocytes.

A comparative evaluation of the PD-L1 expression on TCs and TAICs assessed by standard IHC or 4-Plex IHC by both manual and automated image analysis showed that the automated analysis of the 4-Plex assay correlated well with the manual analysis of either standard IHC (*rho* = 0.85) or 4-Plex assay (*rho* = 0.98; Figure 1 and Figure S1). Thus, the automated 4-Plex signal was preferentially used to further document the impact of the tested multiplex IHC markers.

**Figure 2:**
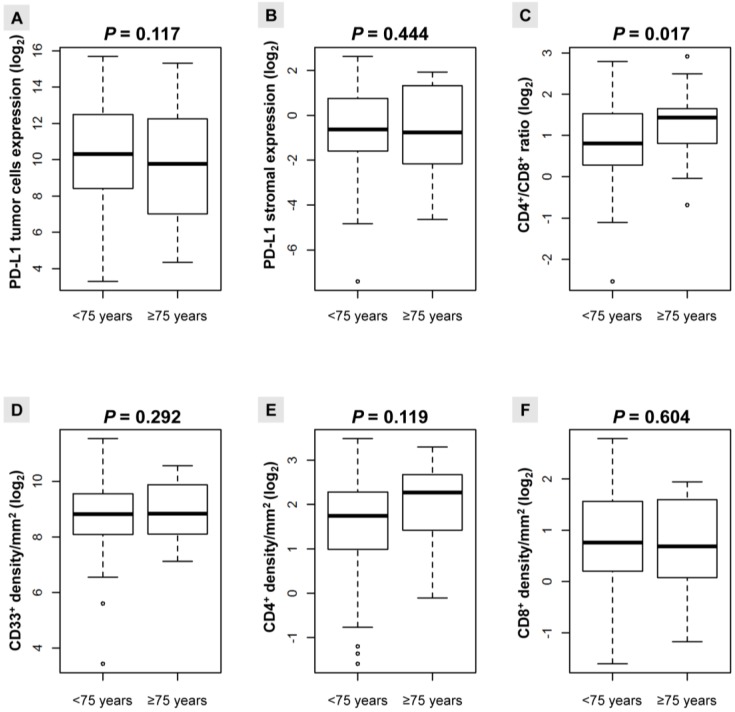
Boxplots for expression (log_2_) of the analyzed biomarkers as continuous variables according to age (<75≤ years). (**A**) Expression of PD-L1 in tumor; (**B**) Expression of PD-L1 in stromal cells; (**C**) CD4^+^/CD8^+^ ratio; (**D**) CD33^+^ density/mm^2^; (**E**) CD4^+^ density/mm^2^; (**F**) CD8^+^ density/mm^2^.

The PD-L1 expression and the density of lymphocyte and MDSC subpopulations reflected the expected incidence according to their phenotype in LADC (Figure 1 and Figure 2) [5]. In addition, no difference was observed in the CD8^+^ lymphocyte density between the two sequential slides from the same case (Figure 1; *p* = 0.964).

### 2.2. Distribution of PD-L1 Expression and the TAICs Density According to Age

When we examined correlation between PD-L1 expression and TAICs density, we found different patterns of correlation according to age. Several cutoffs for patients’ age were evaluated (≥50, ≥55, ≥60, ≥65, ≥70, ≥75, and ≥80 years), with however significant correlation observed only when patients were <75≤ years (Figure 2). The CD4^+^/CD8^+^ ratio was significantly higher in patients ≥75 years (*p* = 0.017). Although individually they failed to meet statistical significance, this pattern may be related to the increase in the CD4^+^ density, and the decrease in the CD8^+^ density in patients ≥75 years (Figure 2). Likewise, there was a non-significant trend towards a decrease in PD-L1 expression in the tumor compartment in patients ≥75 years (Figure 2).

In patients <75 years, the density of TAICs, except for the CD33^+^ density, exhibited a positive significant correlation with expression of PD-L1 in tumor cells, whereas we found a lower correlation of PD-L1 expression in tumor cells with TAICs marker density in elderly patients ≥75 years (Table 1).

By combining the PD-L1 expression in TCs with the density of analyzed TAICs, as proposed by Teng et al. [4], we were able to identify the 4 subtypes of tumor microenvironments in LADC cases according to age (Table 2; Figure S2).

This analysis showed that 36% of patients <75 years and 30% of patients ≥75 years had a type I adaptive immune resistance pattern (defined as positive PD-L1 expression and positive CD8^+^), and that 34% of patients <75 years and 33% of patients ≥75 years had a type II immune ignorance phenotype (defined as a negative CD8^+^ and negative PD-L1 expression). Tumors with type III with intrinsic induction (positive PD-L1 expression without CD8^+^) and type IV immune tolerance (positive CD8^+^ and negative PD-L1 expression) patterns were less frequently detected. A similar distribution in each subgroup was found when the analysis included CD4^+^ and CD33^+^ cells and PD-L1 expression in TAICs. However, while the proportion of subgroups significantly varied in patients <75 years, there was no significant difference in patients ≥75 years, other than PD-L1 expression in stromal cells (Table 2; *p* = 0.001).

Interestingly, a positive CD4^+^/CD8^+^ ratio with (33%) or without (37%) positive PD-L1 expression in tumor cells was more frequently observed in patients ≥ 75 years.

### 2.3. Correlation with Clinicopathological Features and Prognosis of LADC Patients

In the whole study population, the median CD4^+^/CD8^+^ ratio (*p* = 0.032) and the median CD33^+^ density (*p* = 0.008) were significantly associated to the pTNM stage. According to age, no significant correlations were observed between the analyzed markers and the gender, histological subtype, smoking history, *EGFR* or *KRAS* mutation status, and the pTNM stage (Table S1).

**Figure 3:**
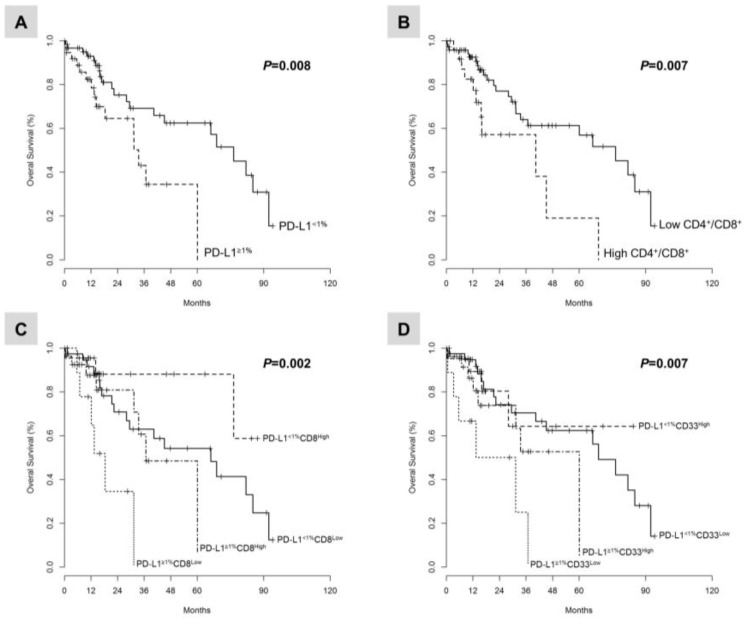
Kaplan-Meier curves illustrate the duration of overall survival according to: (**A**) the PD-L1 expression in TCs; (**B**) the CD4^+^/CD8^+^ ratio; (**C**) the combined analysis of PD-L1 expression in TCs and the CD8^+^ cell density; and (**D**) the combined analysis of PD-L1 expression in TCs and the CD33^+^ cell density. Cutoff values were PD-L1 ≥1% TCs and median for the CD4^+^/CD8^+^ ratio, the CD8^+^ and CD33^+^ cell density, respectively.

In the whole study population, the univariate and multivariate analyses adjusted for pTNM stage (*p* = 0.004; HR, 5.3; 95% confidence interval (CI), 1.7–16) demonstrated that the PD-L1 expression ≥1% tumor cells (*p* = 0.008; HR, 2.6; 95% CI, 1.2–5.4) and a CD4^+^/CD8^+^ ratio higher than the median (*p* = 0.007; HR, 2.7; 95% CI, 1.3–5.8) significantly correlated with poor OS duration (Figure 3), while a CD8^+^ density higher than the median indicated a non-significant trend to improved OS (*p* =0.08; HR, 0.55; 95% CI, 0.27–1.1) (Table S2).

The combination of PD-L1 expression ≥1% tumor cells with a CD8^+^ density lower than the median (*p* = 0.002; HR, 4.2; 95% CI, 1.5–12), or with a CD33^+^ density lower than the median (*p* = 0.007; HR, 4.7; 95% CI, 1.7–13) identified a subset of LADC patients with poor OS (Figure 3, Table S2).

Following these findings, we assessed each marker for its ability to predict OS in patients ≥75 years of age, after adjusting for the pTNM stage. A CD4^+^/CD8^+^ ratio higher than the median (*p* = 0.05; HR, 1.6; 95% CI, 0.4–6.3), the combined PD-L1 expression ≥1% TCs with a low CD8^+^ density (*p* = 0.048; HR, 5.1; 95% CI, 0.8–32), or with a low CD33^+^ density (*p* = 0.046; HR, 6.3; 95% CI, 1–38) identified a subset of elderly LADC patients with poor OS. Moreover, the combined PD-L1 expression ≥1% TCs with a high CD4^+^ density (*p* = 0.043; HR, 7.2; 95% CI, 1.1–18) was associated with poor OS. Finally, PD-L1 expression ≥1% TCs alone did not affect OS duration in elderly patients (*p* = 0.137; HR, 2.7; 95% CI, 0.69–11), but did in patients <75 years (*p* = 0.012; HR, 3.1; 95% CI, 1.2–7.9; Table S2).

## 3. Discussion

In this study, we investigated PD-L1 expression in tumors and TAICs, as well as the immune characteristics of whole tumor sections from tumors of patients with LADC stratified by age. We used a multiplex IHC methodology optimized for immune complexity associated with quantitative studies using a computational image processing workflow.

We identified distinct environmental patterns in tumors according to the patients’ age, and observed that several single or combined markers correlated with the outcome of LADC patients.

Elderly patients over 75 years with LADC demonstrated a significantly higher CD4^+^/CD8^+^ ratio, potentially based on an accumulation of CD4^+^ cells and on a decrease in CD8^+^ cell density. We found a non-significant trend towards lower incidence of PD-L1 expression in both tumor and stromal compartments, as well as a decreased density of CD33^+^ cells in elderly patients. Interestingly, PD-L1 expression in TCs correlated less with the CD4^+^ and CD8^+^ TAICs density and PD-L1 expression in stromal cells in elderly patients ≥75 years than in patients <75 years. While different immune cells such as CD8^+^ and CD4^+^ cells are capable of inducing PD-L1 expression in TCs through IFNγ production, our findings suggest that these mechanisms might be less active in elderly patients [14,15]. Thus, for the first time, to the best of our knowledge, the multiplex IHC platform on FFPE tumor samples affirmed the presence of less effective anti-tumor immunoreactivity in elderly patients, as previously demonstrated by flow cytometry analysis of blood or fresh tumor samples [16,17].

In our study, we characterized according to age the 4 types of tumor microenvironments described by Teng et al. using as criteria each immune marker combined with the PD-L1 expression in TCs [4]. In elderly patients, we found that type I LADC with adaptive immune resistance (defined as positive PD-L1 and positive CD8^+^),most likely responsive to checkpoint blockade, was less frequent than in patients <75 years (30% vs. 36%), in contrast to type III (positive PD-L1 and negative CD8^+^); this highlights the fact that PD-L1 positivity alone cannot be taken as a predictive factor for response to anti-PD-1/PD-L1 therapies in elderly patients. Moreover, for 19% of LADC patients ≥75 years with a type IV microenvironment, other immunosuppressive pathways may dominate, such as those linked to MDSCs [18]. In our study, however, the CD33^+^ cell density did not change with age, while a high level of CD33^+^ infiltration combined with expression of PD-L1 <1% TCs significantly correlated with better OS in elderly patients, indicating a secondary role of CD33^+^ cells in limiting cancer-promoting inflammation and tumor growth [19]. Additional markers such as CD66b, CD14, CD11b, and CD15 should be investigated to capture the phenotype complexity of MDSCs [20].

A key understudied issue in NSCLC is the potential for PD-L1 expression and TAIC density to predict outcome and response to PD-1/PD-L1 inhibitors in elderly patients. Our data point to at least two distinct pathways leading to worse outcome of LADC in elderly patients: (1) a shift in the CD4/CD8 T-cell ratio, and (2) PD-L1 expression ≥1% tumor cells combined with a low CD8^+^ or CD33^+^ cell density and a high CD4^+^ cells.

CD4^+^ cells are generally considered to be immunosuppressive and have been linked to poor outcome in several types of solid tumors, including NSCLC [21,22,23]. Some studies have shown that the CD4^+^/CD8^+^ ratio may give more prognostic information than either parameter alone. In particular, a high CD4^+^/CD8^+^ ratio has been associated with poor outcome in colorectal carcinoma and glioma, and favorable disease outcome in mesothelioma patients [23,24,25]. Our results showed that a high CD4^+^/CD8^+^ ratio has been associated with poor disease outcome in the whole population, as well as in elderly patients, whereas the CD4^+^ or CD8^+^ cell density alone did not significantly affect the OS duration. 

Moreover, the type III microenvironment, characterized by positive PD-L1 expression in TCs and a low CD8^+^ density, was associated with poor OS in elderly patients. It is noteworthy that PD-L1 expression alone was associated with disease outcome in patients <75 years, but not in elderly patients. These results suggest that the association of CD8^+^ cells with PD-L1 expression may be more meaningful than PD-L1 expression alone for prediction of survival in elderly patients. In addition, patients with PD-L1-positive tumors accompanied by a high density of CD8^+^ cells might achieve a better outcome through blockade of the PD-1/PD-L1 pathway [26]. Further studies are warranted to clarify the association between PD-L1 expression and TAICs, and to determine whether this combination has predictive relevance as a biomarker for selecting elderly individual patients for treatment involving PD-1/PD-L1 blockade.

Only a few studies have reported some differences in density of TAICs among a broad range of age groups in NSCLC [27,28]. However, these studies were not designed with the goal of evaluating PD-L1 expression and the immune cell infiltration with particular interest in elderly patients. Only one study investigated seven immune markers in a large cohort of octogenarians with NSCLC, and reported similar rates of tumor immune cell infiltration between elderly and younger patients [29]. Of the examined immune markers, only the presence of a low level of tumor infiltration of CD68^+^ cells in the octogenarian age subgroup correlated with an increased risk of recurrence. However, this latter study contained several limitations: (1) the selection of patients exclusively with stage I NSCLC; (2) the use of tissue microarrays, which will likely affect the accuracy needed for the characterization of various immune components, given that many tumors are heterogeneous with respect to the proportion of lymphoid and myeloid cells [30]; and (3) the absence of a scoring system and cutoffs for positivity. Notably, immune infiltrates are highly heterogeneous, not only between tumor types, but also within a tumor or between different patients with the same cancer types [4]. Thus, whereas sequential IHC may not be able to assess single-cell-based immune phenotypes, the approach described herein circumvents these issues.

In our study, we analyzed immune markers using a new emerging technique for the detection of multiple biomarkers within a single tissue section [31]. The brightfield chromogenic multiplex IHC methodology used herein was able to unmix four dyes while preserving the tissue morphology and architecture. In most studies using multiplexed imaging approaches to examine the immune features of tumor microenvironment, multiplex fluorescence IHC was used [28,32,33]. Although this approach offers insight into cellular and molecular mechanisms, several challenges have been described in current practice, such as the difficulty of interpretation, multiple fluorophores blending together complicating resolution and thus muddling visual assessment, and the potential for tissue autofluorescence in FFPE samples, further complicating visual interpretation [31].

Moreover, semi-quantitative PD-L1 and TAIC density scoring systems on the basis of microscopic observation of slides have been used mostly [34,35]. Visual examination of IHC stained tissue sections remains a subjective process characterized by some intraobserver and interobserver variability and reduced reproducibility, which can be overcome by digital image analysis [5,36,37]. Our workflow increased pathologist accuracy by automatically measuring parameters which are hard to achieve reliably by eye, such as the PD-L1 expression in tumor and stromal cells [37].

However, the present study suffered from several limitations imposed by its retrospective design in a single institution, the limited number of patients with available tumor tissue, as well as the small number of elderly patients and lack of immunotherapy regimens. The functionality of infiltrating immune cells and effect of other relevant immune cells was not assessed, and further experiments using cell lines and animal models are warranted [38,39]. Moreover, our choice for the selected markers was limited by the current development of the brightfield multiplex IHC technology, which does not allow mixing of more than 4 or 5 chromogens, in contrast to a fluorescence multiplex approach, which allows mixing of up to 12 fluorophores [31]. The analysis of other markers of interest such as PD-1, FoxP3, CD14, or CD11b should be considered. In addition, further analysis in a larger population of elderly patients treated with anti-PD-1/PD-L1 agents is required.

Nevertheless, with recent results emphasizing the role of TAICs as a critical parameter in predicting the efficacy of anti-PD-1/PD-L1 inhibitors [40], our approach integrating brightfield chromogenic multiplex IHC with digital image analysis may be transferred into clinical pathology practice, provided the use of simple algorithms and independent validation on a larger population, and may certainly serve to further improve the understanding of PD-1/PD-L1 immune checkpoint targeting.

## 4. Materials and Methods

### 4.1. Patients

This retrospective cohort consisted of 101 patients stratified by age (<75≤ years) with surgically treated LADC at the Department of Thoracic Surgery, University Côte d’Azur, Pasteur Hospital (Nice, France), from March 2010 to October 2016. Tumor specimens were collected, stored, and used with informed consent from the patients (Hospital-Integrated Biobank BB-0033-00025, Pasteur Hospital, Nice, France). The study was approved by the Ethics Committee of the University Côte d’Azur and performed according to the guidelines of the Declaration of Helsinki (04-APN-17; 2 March 2017). The study complied with the REMARK recommendations for tumor marker prognostic studies using biological material [41]. The criteria to select samples were: tumor cell content of more than 50%, availability of whole tissue sections, resectable disease with no neoadjuvant treatment, and lack of any infection, autoimmune disease or corticosteroid therapy 6 months before surgery. The main clinical and histomolecular characteristics of the patients are summarized in Table 3.

Targetable genomic alterations were evaluated by pyrosequencing (therascreen *EGFR/KRAS/BRAF* Pyro Kit, Qiagen, Hilden, Germany) or by FISH (Vysis LSI *ALK* Dual Color, Abbott Molecular Inc., Des Plaines, IL, USA; and Kreatech^TM^ *ROS1* 6q22 Break Fish Probe, Leica Biosystems, Amsterdam, The Netherlands). Tumors expressed no genomic alterations in the *BRAF*, *ALK* and *ROS1* genes.

### 4.2. Multiplex Immunohistochemistry 

IHC staining was performed on the Discovery Ultra automated immunostainer (Ventana Medical Systems, Tucson, AZ, USA). The multiplex technology uses sequential application of unmodified primary antibodies with a specific Heat Deactivation steps in between that does not impact epitope in the tissue (Figure S3) [42].

In a sequential staining procedure, deactivation of the primary antibody and secondary antibody-HRP/AP bound to the first biomarker, prior to the application of subsequent biomarker(s), is critical to reducing cross-reactivity and facilitating downstream image analysis. The Cell Conditioning 2 buffer (CC2, #950-123, Ventana, Tucson, AZ, USA) was used for deactivation of the bound primary antibody and secondary antibody-HRP, while maintaining the integrity of the tissue morphology and the subsequent epitopes. Deparaffinization and on-board antigen retrieval were performed for 40 min at 100 °C with the CC1 reagent (#950-500, Ventana). Two slides were colored using VENTANA reagents except as noted, according to the manufacturer’s instructions.

On the first slide, four pre-diluted primary antibodies (4-Plex) were sequentially applied in the following order using the indicated chromogenic detection: rabbit anti-PD-L1 (clone SP263, #741-4905) with Discovery Teal-HRP (#760-247), rabbit anti-CD8 (clone SP57, #790-4460) with Discovery Purple (#760-229), rabbit anti-CD33 (clone SP266, #760-4952) with ChromoMapDAB (#760-159) and mouse anti-Pan Keratin AE1/AE3 (clone PCK26, #760-2135) with Discovery Yellow AP (#760-239) (Figure S3). On the second slide, two pre-diluted primary antibodies (2-Plex) were applied: rabbit anti-CD8 (clone SP57, #790-4460) with Discovery Teal-HRP (Beta test reagent), and rabbit anti-CD4 (clone SP35, #790-4423) with Discovery Purple (#760-229). Finally, the slides were counterstained with hematoxylin and bluing reagent.

Two different controls for the staining method were applied: (1) a blank control by omission of the primary antibody in every sequence of staining, and (2) tonsil positive tissue control to verify the specificity of the staining for every staining procedure.

### 4.3. Image Acquisition and Automated Digital Quantification

Whole stained slides were scanned using the Nanozoomer HT 2.0 scanner (Hamamatsu Photonics, Hamamatsu, Japan). Senior thoracic pathologists screened each slide under a microscope, and selected one large intratumoral representative region of interest (median surface, 26 mm^2^) within the intact tumor area (at least 50% of TCs) lacking necrosis. For automated digital quantification, CaloPix (TRIBVN Healthcare, Châtillon, France) algorithms were used (Figure S3) [43]. The first step was to identify the tissue by removing all white pixels from the background. This step was performed using a morphometric algorithm by thresholding the gray level intensity image. The second step was to quantify the different biomarkers in the image. We used two analytical algorithms depending on the cell staining, both of which were based on a machine learning approach: (i) for well-defined and homogeneous cell architectures such as for CD33 stained cells, we used the algorithm called “Immuno object by learning” of CaloPix, which points out every single cell in the analytical region, and thus, gives the total number of cells, and (ii) for heterogeneous and poorly-separated cells such as PD-L1 cells, we used the “tissue recognition” algorithm, which gives the surface of the desired stain. 

The main steps of the required quantifications were the following: (i) CD33 quantification by a morphometric analysis for the tissue determination followed by an “Immuno object by learning” for cell detection and counting, (ii) PD-L1 quantification by using morphometry analysis for tissue determination followed by “tissue recognition” for tumor and stroma identification in which the yellow color was isolated by a color unmixing process, and secondary tissue recognition was used afterwards to identify the PD-L1 staining by extracting the green color, and (iii) CD4 and CD8 quantification by using morphometry analysis for the tissue determination followed by “tissue recognition” for CD4 identification by extracting the purple color and another “tissue recognition” for CD8 identification by extracting the blue color.

The density of TAICs expressing CD8, CD4, and CD33 was divided on the basis of regular values of distribution by the statistical software, and the median density was considered positive [4,5]. PD-L1 expression was analyzed either as a continuous variable, or based on a clinical validated threshold ≥1% of tumor cells.

Two different controls for the quantification method were applied: (1) manual and automated analysis of the PD-L1 staining assessed by 4-Plex IHC and by a standard IHC assay, and (2) automated analysis of the CD8 staining on each of the two slides per case to assess the reproducibility of the staining between slides.

### 4.4. Statistical Analysis

Correlation between the results of the PD-L1 IHC assays (manual vs automated) was determined by calculating the Spearman’s rank correlation coefficient (*rho*) and computing the Bland-Altman agreement plots. The Wilcoxon test was used to detect differences in continuous variables between groups of patients, given that the distribution of data was not normal (Kolmogorov-Smirnov test). Kaplan-Meier survival curves, log-rank test and Cox proportional regression analysis were determined to assess the prognostic significance of the tested markers for OS. All statistical analyses and data presentations were performed in R language (version 3.2.2, R Core Team, Vienna, Austria). All statistical tests were 2-sided, and *p*-values < 0.05 indicated statistical significance.

## 5. Conclusions

In summary, this study demonstrated that the tumor immune microenvironment in elderly patients with LADC is different from that of younger patients, thereby bearing prognostic implications. With an increasing elderly population, these present findings provide insight into the complexity of the immune microenvironment of this subgroup of patients, and may help clinicians to better stratify elderly patients with LADC for immunotherapy.

## Figures and Tables

**Table 1:** Correlation between the PD-L1 expression in tumor cells and the TAICs density by mm^2^ according to the age of patients with LADC.

Immune Marker	PD-L1 Expression in Tumor Cells			
	<75 Years		≥75 Years	
	*rho*	*p*-Value *	*rho*	*p*-Value *
CD4	0.61	<0.001	0.38	0.001
CD8	0.60	<0.001	0.50	<0.001
PD-L1 in stromal cells	0.86	<0.001	0.79	0.001
CD33	0.19	0.342	0.41	<0.001

* Spearman correlation test.

**Table 2:** Distribution of the types of microenvironment in LADC age-stratified specimens based on the PD-L1 expression in tumor cells or in immune cells, and on the density of TAICs. The Fisher’s exact test was used.

TAICs Density	Age < 75 Years			Age ≥ 75 Years		
	PD-L1 Expression in Tumor Cells Negative	PD-L1 Expression in Tumor Cells Positive	*p*-Value	PD-L1 Expression in Tumor Cells Negative	PD-L1 Expression in Tumor Cells Positive	*p*-Value
**CD8^+^ cells**			< 0.001			0.256
Negative	25 (34%)	11 (15%)	-	9 (33%)	5 (19%)	-
Positive *	11 (15%)	26 (36%)	-	5 (19%)	8 (30%)	-
**CD4^+^ cells**			0.004			0.236
**Negative**	26 (36%)	14 (19%)	-	7 (26%)	3 (11%)	-
Positive *	10 (13%)	23 (32%)	-	7 (26%)	10 (37%)	-
**CD4^+^/CD8^+^ ratio**			0.814			0.999
Negative	20 (27%)	22 (30%)	-	4 (15%)	4 (15%)	-
Positive	16 (22%)	15 (21%)	-	10 (37%)	9 (33%)	-
**CD33^+^ cells**			0.019			0.695
Negative	23 (32%)	13 (18%)	-	8 (30%)	5 (19%)	-
Positive *	13 (18%)	24 (33%)	-	6 (25%)	7 (26%)	-
**PD-L1 stromal cells**			< 0.001			0.001
Negative	30 (41%)	5 (7%)	-	12 (44%)	2 (7%)	-
Positive *	6 (8%)	32 (44%)	-	2 (7%)	10 (37%)	-

* Median PD-L1 expression in tumor cells and median TAICs determined by CD8, CD4, PD-L1 stromal, and CD33 cell densities were defined as positive.

**Table 3:** Clinical and histopathological characteristics of the 101 LADC patients included in the study.

Feature	Overall *n* (%)
Patient number	101 (100%)
Age (years)	
<75 years	74 (73%)
≥75 years	27 (27%)
Gender	
Male	61 (60%)
Female	40 (40%)
Smoking history	
Never smoked	17 (17%)
Former or current smokers	84 (83%)
Histological pattern	
Acinar	53 (52%)
Solid	19 (19%)
Papillary	13 (13%)
Lepidic	9 (9%)
Micropapillary	7 (7%)
TNM stage	
II	66 (65%)
III	16 (16%)
IV	19 (19%)
*EGFR* status	
Mutant	7 (7%)
Wild-type	94 (93%)
*KRAS* status	
Mutant	21 (21%)
Wild-type	80 (79%)

Abbreviations: TNM: tumor, node, metastasis.

## Supplementary Materials

The following are available online at http://www.mdpi.com/2072-6694/10/9/326/s1, Figure S1: Development of the M-Plex IHC and image quantification platform; Figure S2: Comparative analysis of PD-L1 expression in tumor cells assessed by automated or manual analysis of standard or 4-Plex IHC protocols; Figure S3: Distribution of the 4 types of tumor microenvironment described by Teng et al. [4] according to age and selected markers; Table S1: Correlation between PD-L1 expression in tumor and stromal cells (≥1%) and median density by mm^2^ of TAICs expressing immune markers and clinicopathological features in LADC specimens according to age; Table S2. Explanatory prognostic factors for overall survival in a Cox proportional hazards model.
