# Supplementary material for: Chromogenic Multiplex Immunohistochemistry Reveals Modulation of the Immune Microenvironment Associated with Survival in Elderly Patients with Lung Adenocarcinoma
Source: Cancers (Basel). 2018 Sep 13;10(9):326. doi: 10.3390/cancers10090326 (PMC6162494; doi:10.3390/cancers10090326)
Supplement: Supplementary file 1 [file cancers-10-00326-s001.pdf]

Supplementary Materials: Chromogenic Multiplex Immunohistochemistry Reveals Modulation of the Immune Microenvironment Associated with Survival in Elderly Patients with Lung Adenocarcinoma

Marius Ilié, Mélanie Beaulande, Saima Ben Hadj, Emmanuel Chamorey, Renaud Schiappa, Elodie Long-Mira, Sandra Lassalle, Catherine Butori, Charlotte Cohen, Sylvie Leroy, Olivier Guérin, Jérôme Mouroux, Charles-Hugo Marquette, Jean-François Pomerol, Gilles Erb, Véronique Hofman, Paul Hofman

For well-defined and homogeneous cell architectures such as the CD33 stained cells, we used the algorithm called "Immuno object by learning" of CaloPix which points out every single cell in the analytical region and thus gives the total number of cells.

For heterogeneous and poorly-separated cells such as PD-L1 cells, we used the "tissue recognition" algorithm, which gives the surface of the desired stain.

In both cases, the identification of the cell/tissue was performed using a probabilistic classification approach based on machine learning. The desired stain was first isolated using a color unmixing process. Different classes including the cell class and the non-cell class(es) were defined on the unmixed image and a set of discrimination criteria based on the color, texture, and edge were computed. These criteria are learned for each class from a given example region. A decision model called random forest tree (RFT) was then created and then applied to any new image giving a probability map belonging to the studied class. The probability map was then filtered to get the surface of the cell class or post-processed to point out the cell centers.

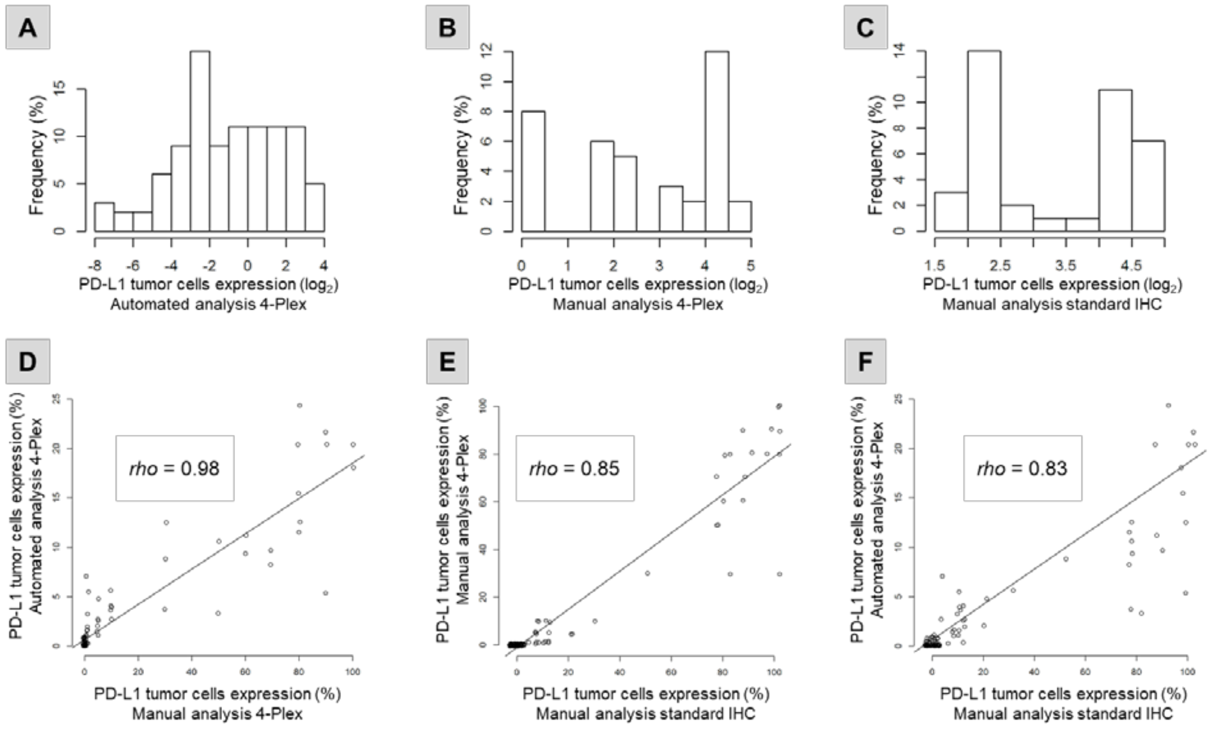

Figure S1. Comparative analysis of PD-L1 expression in tumor cells assessed by automated or manual analysis of standard or 4-Plex IHC protocols. (**A**–**C**) Range of log2 PD-L1 TCs expression according to the type of analysis and IHC protocols. (**D**–**E**) Scatter plots and Spearman correlation coefficients (rho) of PD-L1 measures according to the type of analysis and IHC protocols.

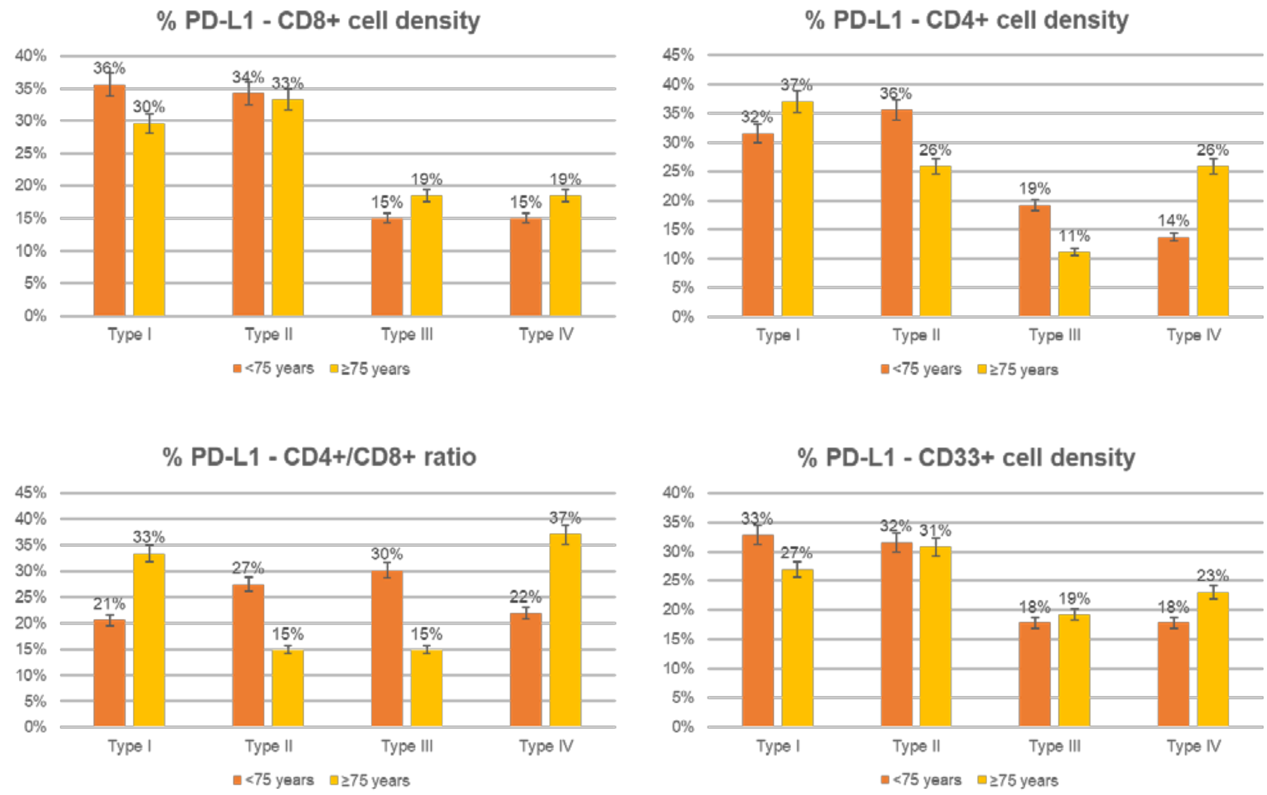

Figure S2. Distribution of the 4 types of tumor microenvironment described 39 by Teng et al. [1] according to age and selected markers.

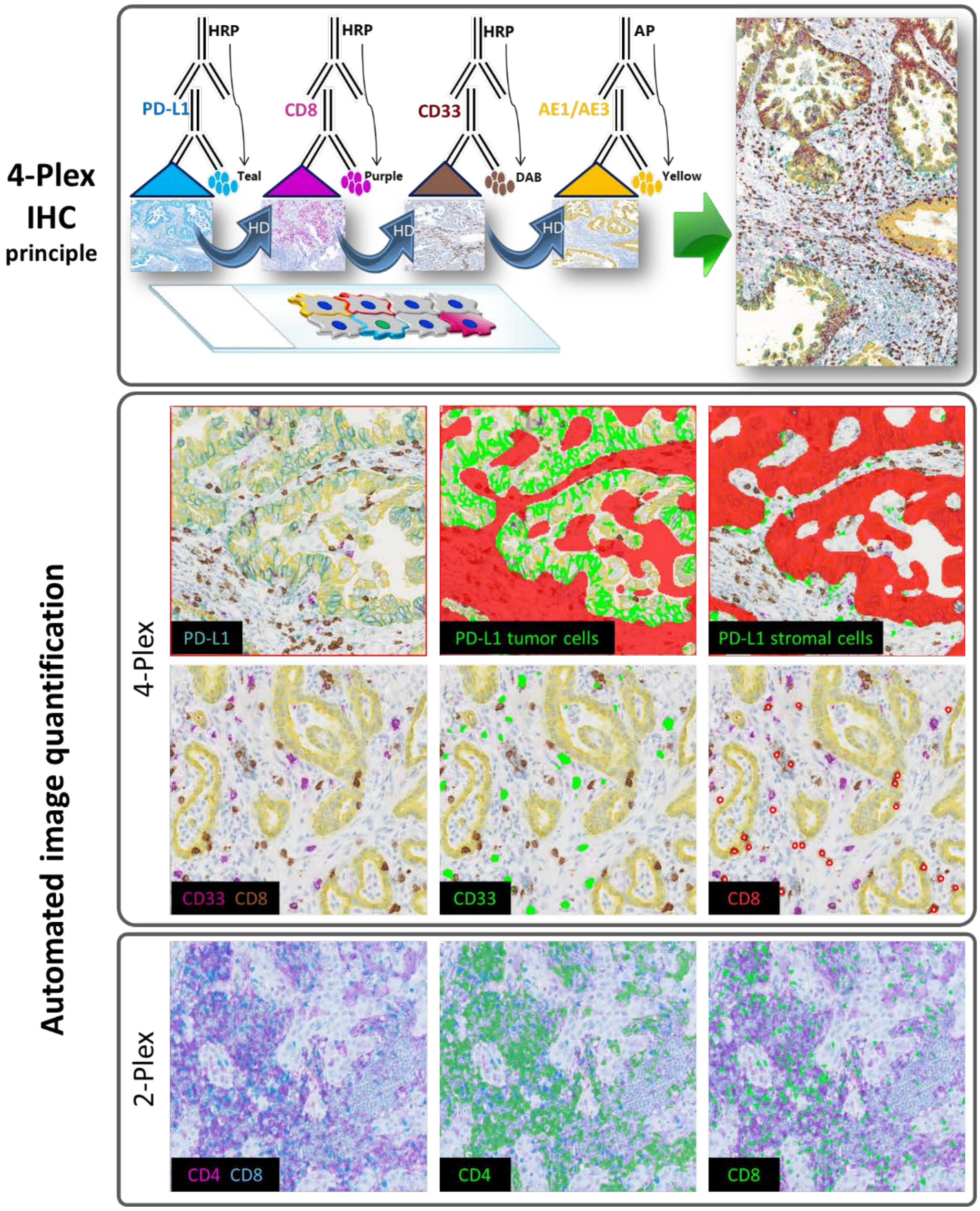

Figure S3. Development of the M-Plex IHC and image quantification platform. Upper panel: Description of the 4-Plex IHC principle. The multiplexing technology was performed on the Discovery Ultra automated immunostainer (Ventana Medical Systems, Tucson, AZ) and used sequential application of unmodified primary antibodies with, among each, a specific Heat Deactivation (HD) step that does not impact on the epitope in the tissue. Lower panel: Automated image quantification of the two IHC protocols used in the study. We used two analytical algorithms depending on the cell staining, both based on a machine learning approach. Original magnification x200. Chromogenic colors: Teal, PD-L1; Purple, CD8; CD33, brown; AE1/AE3, yellow; blue, hematoxylin counterstain.

Table S1. Correlation between PD-L1 expression in tumor and stromal cells (≥1%) and median density by mm² of TAICs expressing immune markers and clinicopathological features in LADC specimens according to age.

(A). Patients < 75 years

Analyzed markers	Smoking history			Histological subtype			pTNM stage			*EGFR* status			*KRAS* status		
	**Never**	**Current /former**	***p***	**Solid**	**Non-solid**	***p***	**Early I+II**	**Late III+IV**	***p***	**WT**	**Mutation**	***p***	**WT**	**Mutation**	***p***
**PD-L1 tumor cells**			0.245			0.516			0.804			1			0.426
Negative	7 (87%)	39 (59%)		37 (60%)	9 (75%)		30 (64%)	16 (59%)		18 (72%)	3 (75%)		11 (79%)	9 (60%)	
Positive*	1 (13%)	27 (41%)		25 (40%)	3 (25%)		17 (36%)	11 (41%)		7 (28%)	1 (25%)		3 (21%)	6 (40%)	
**PD-L1 stromal cells**			0.464			0.508			0.805			1			0.054
Negative	6 (75%)	38 (58%)		36 (58%)	8 (67%)		29 (62%)	15 (56%)		16 (64%)	3 (75%)		11 (79%)	7 (47%)	
Positive*	2 (25%)	27 (42%)		26 (42%)	3 (25%)		18 (38%)	11 (41%)		8 (32%)	1 (25%)		2 (14%)	8 (53%)	
**$CD8^+$ cells**			0.479			1			0.629			0.479			0.065
Negative	5 (63%)	31 (47%)		30 (48%)	6 (50%)		24 (51%)	12 (44%)		12 (48%)	4 (100%)		10 (72%)	5 (33%)	
Positive*	3 (37%)	34 (53%)		31 (50%)	6 (50%)		22 (47%)	15 (56%)		13 (52%)	0 (0%)		4 (29%)	10 (67%)	
**$CD4^+$ cells**			0.280			1			0.467			0.121			0.263
Negative	6 (75%)	34 (53%)		33 (53%)	7 (58%)		27 (58%)	13 (48%)		13 (52%)	4 (100%)		10 (71%)	7 (47%)	
Positive*	2 (25%)	31 (47%)		28 (45%)	5 (42%)		19 (41%)	14 (52%)		12 (48%)	0 (0%)		4 (29%)	8 (53%)	
**$CD4^+/CD8^+$ ratio**			0.715			1			0.811			0.104			1
Negative	4 (50%)	38 (58%)		35 (56%)	7 (58%)		27 (58%)	15 (56%)		18 (72%)	1 (25%)		9 (64%)	10 (67%)	

Positive	4 (50%)	27 (42%)		26 (42%)	5 (42%)		19 (41%)	12 (44%)		7 (28%)	3 (75%)	1	5 (36%)	5 (33%)	
**CD33⁺ cells**			0.479			0.344			0.807						1
Negative	5 (63%)	31 (47%)		29 (47%)	7 (58%)		24 (51%)	12 (44%)		15 (60%)	2 (50%)		7 (50%)	9 (60%)	
Positive*	3 (37%)	34 (53%)		33 (53%)	4 (33%)		23 (49%)	14 (52%)		9 (36%)	2 (50%)		6 (43%)	6 (40%)	

(B). Patients ≥ 75 years

**Analyzed markers**	**Smoking history**			**Histological subtype**			**pTNM stage**			***EGFR* status**			***KRAS* status**		
	**Never**	**Current / former**	***p***	**Solid**	**Non-solid**	***p***	**Early I+II**	**Late III+IV**	***p***	**WT**	**Mutation**	***p***	**WT**	**Mutation**	***p***
**PD-L1 tumor cells**			0.667			1			0.665			1			1
Negative	6 (75%)	11 (61%)		16 (62%)	1 (100%)		11 (58%)	6 (75%)		8 (80%)	3 (100%)		6 (86%)	5 (83%)	
Positive*	2 (25%)	7 (39%)		10 (39%)	0 (0%)		8 (42%)	2 (25%)		2 (20%)	0 (0%)		1 (14%)	1 (17%)	
**PD-L1 stromal cells**			1			1			0.394			1			1
Negative	5 (63%)	10 (56%)		14 (54%)	1 (100%)		9 (48%)	6 (75%)		8 (80%)	2 (67%)		5 (71%)	5 (83%)	
Positive*	3 (37%)	7 (39%)		11 (42%)	0 (0%)		9 (48%)	2 (25%)		2 (20%)	1 (33%)		2 (29%)	1 (17%)	
**CD8⁺ cells**			0.682						1			0.496			0.265
Negative	5 (63%)	9 (50%)		13 (50%)	1 (100%)		10 (53%)	4 (50%)		6 (60%)	3 (100%)		6 (86%)	3 (50%)	
Positive*	3 (37%)	9 (50%)		13 (50%)	0 (0%)		9 (47%)	4 (50%)		4 (40%)	0 (0%)		1 (14%)	3 (50%)	
**CD4⁺ cells**			1			1			0.414			0.510			1
Negative	3 (37%)	7 (39%)		10 (38%)	0 (0%)		6 (32%)	4 (50%)		7 (70%)	1 (33%)		4 (57%)	4 (67%)	
Positive*	5 (63%)	11 (61%)		16 (62%)	1 (100%)		13 (68%)	4 (50%)		3 (30%)	2 (67%)		3 (43%)	2 (33%)	
**CD4⁺/CD8⁺ ratio**			1			1			0.182			1			0.286

Negative	2 (25%)	6 (33%)		8 (31%)	0 (0%)		4 (21%)	4 (50%)		5 (50%)	1 (33%)		2 (29%)	4 (67%)	
Positive	6 (75%)	12 (67%)		18 (69%)	1 (100%)		15 (79%)	4 (50%)		5 (50%)	2 (67%)		5 (71%)	2 (33%)	
**$CD33^+$ cells**			0.096			1			0.672			0.192			0.129
Negative	6 (75%)	6 (33%)		12 (46%)	1 (100%)		10 (53%)	3 (38%)		4 (40%)	3 (100%)		6 (86%)	1 (17%)	
Positive*	2 (25%)	11 (61%)		13 (50%)	0 (0%)		8 (42%)	5 (62%)		6 (60%)	0 (0%)		1 (14%)	5 (83%)	

Table S2. Explanatory prognostic factors for overall survival in a Cox proportional hazards model.

Prognostic factors	Univariate analysis			Multivariate analysis [a]		
	HR	95% CI	*p*	HR	95% CI	*p*
**Whole population**						
Gender	1.2	0.58–2.4	0.645	-	-	-
Smoking history	0.48	0.22–1	0.619	-	-	-
pTNM stage	5.3	1.7–16	0.004	3.3	1.1–5.8	0.008
PD-L1 TCs	2.6	1.2–5.4	0.008	2.3	1–5.1	0.037
PD-L1 TCs and ICs	0.32	0.043–2.4	0.270	-	-	-
CD4	1.1	0.54–2.3	0.776	-	-	-
CD8	0.55	0.27–1.1	0.080	0.45	0.19–1.06	0.064
CD4/CD8	2.7	1.3–5.8	0.007	2.4	1–5.8	0.033
PD-L1 TCs/CD8	4.2	1.5–12	0.002	3.8	1.1–5.6	0.035
PD-L1 TCs/CD4	0.48	0.14–1.7	0.251	-	-	-
PD-L1 TCs/CD33	4.7	1.7–13	0.007	2.8	1–4.8	0.043
**Patients < 75 years**						
Gender	1.1	0.54–2.3	0.774	-	-	-
Smoking history	1.3	0.59–2.8	0.536	-	-	-
pTNM stage	2.1	1–4.5	0.049	2.3	1–5	0.041
PD-L1 TCs	3.1	1.2–7.9	0.012	1.2	1–2.1	0.048
PD-L1 TCs and ICs	1.3	0.57–2.9	0.541	-	-	-
CD4	1.7	0.84–3.3	0.145	-	-	-

CD8	0.73	0.32–1.6	0.438	-	-	-
CD4/CD8	3.6	1.3–9.9	0.016	2.7	1.1–6.7	0.031
PD-L1 TCs/CD8	7.7	2.2–26	0.001	4.3	1.7–15	0.008
PD-L1 TCs/CD4	2.7	0.98–7.7	0.054	1.6	0.9–2.9	0.097
PD-L1 TCs/CD33	4	1–16	0.046	1.8	0.9–3.1	0.054
**Patients ≥ 75 years**						
Gender	1.3	0.68–2.5	0.422	-	-	-
Smoking history	0.83	0.35–1.9	0.665	-	-	-
pTNM stage	2.2	1.1–3.5	0.024	2	1–3.8	0.039
PD-L1 TCs	2.7	0.69–11	0.137	-	-	-
PD-L1 TCs and ICs	1.4	0.4–3.9	0.542	-	-	-
CD4	1.2	0.5–3	0.651	-	-	-
CD8	0.68	0.27–1.7	0.409	-	-	-
CD4/CD8	1.6	0.4–6.3	0.050	1.2	0.7–1.9	0.565
PD-L1 TCs/CD8	5.1	0.8–32	0.048	1.3	0.8–2.2	0.262
PD-L1 TCs/CD4	7.2	1.1–18	0.043	1.8	0.6–5.3	0.267
PD-L1 TCs/CD33	6.3	1–38	0.046	1.3	0.4–3.9	0.633

Note: Gender (male = 0, female = 1), smoking status (0 = history negative for smoking, 1 = history positive for smoking), pTNM stage (I+II = 0, III+IV = 1), PD-L1 expression in tumor cells (TCs) (<1% TCs = 0, ≥1% TCs = 1), PD-L1 expression in TCs and ICs (<1% TCs and ICs = 0, ≥1% TCs and ICs = 1), CD4 (< median = 0, ≥ median = 1), CD8 (< median = 0, ≥ median = 1), CD4/CD8 (< median = 0, ≥ median = 1), CD33 (< median = 0, ≥ median = 1). [a] Multivariate analysis is carried out on statistically significant parameters obtained from the univariate model.

Reference

1. Teng, M.W.; Ngiow, S.F.; Ribas, A.; Smyth, M.J. Classifying cancers based on t-cell infiltration and pd-l1. *Cancer Res.* **2015**, *75*, 2139–2145.
